# Apoptotic Sphingolipid Ceramide in Cancer Therapy

**DOI:** 10.1155/2011/565316

**Published:** 2011-01-13

**Authors:** Wei-Ching Huang, Chia-Ling Chen, Yee-Shin Lin, Chiou-Feng Lin

**Affiliations:** ^1^Institute of Basic Medical Sciences, College of Medicine, National Cheng Kung University, Tainan 701, Taiwan; ^2^Institute of Clinical Medicine, College of Medicine, National Cheng Kung University, Tainan 701, Taiwan; ^3^Department of Microbiology and Immunology, College of Medicine, National Cheng Kung University, Tainan 701, Taiwan

## Abstract

Apoptosis, also called programmed cell death, is physiologically and pathologically involved in cellular homeostasis. Escape of apoptotic signaling is a critical strategy commonly used for cancer tumorigenesis. Ceramide, a derivative of sphingolipid breakdown products, acts as second messenger for multiple extracellular stimuli including growth factors, chemical agents, and environmental stresses, such as hypoxia, and heat stress as well as irradiation. Also, ceramide acts as tumor-suppressor lipid because a variety of stress stimuli cause apoptosis by increasing intracellular ceramide to initiate apoptotic signaling. Defects on ceramide generation and sphingolipid metabolism are developed for cancer cell survival and cancer therapy resistance. Alternatively, targeting ceramide metabolism to correct these defects might provide opportunities to overcome cancer therapy resistance.

## 1. Introduction

Apoptosis, also named programmed cell death, is a normal component for cellular homeostasis involving embryonic/organ development and health in human. For tumorigenesis, oncogenic factors are generally involved in activation of antiapoptotic signaling pathways, whereas tumor suppressor factors are normally proapoptotic [1]. During the past two decades, studies of sphingolipids reveal the important role of bioactive sphingolipids, such as ceramide, in regulation of multiple biological functions especially in apoptosis [2–6]. The cytopathic effects of ceramide are proapoptotic as well as necroticlike, depending on the cell types and the dosages of stimulation. Thus, apoptotic signaling caused by ceramide is diverse because several intracellular organelles are generally involved [7]. Inhibiting cell death by interference on ceramide signaling is a key strategy for tumorigenesis escape from apoptotic stimuli. Therefore, for the development of cancer therapy, ceramide metabolic pathways become candidate target currently [8–11].

The antiproliferative activities of ceramide for cancer therapy depend on the induction of various apoptotic pathways as demonstrated previously [12–14]. Most of these studies are based on the exogenous administration of ceramide analogue, particularly C2- and C6-ceramide. Endogenous generation of ceramide through the newly *de novo* synthesis or the hydrolysis of sphingomyelin is also reported to trigger signaling pathways after apoptotic stimulation. However, it remains controversial for verifying the different molecular mechanisms between these two experimental approaches. In this article, we briefly discussed the link of ceramide and organelle dysfunction in apoptosis and also summarized several ceramide-based mechanisms of cancer therapy resistance as well as strategies by targeting ceramide metabolism for cancer therapy sensitization. 

## 2. Apoptotic Signaling through the Multiple Intracellular Organelle Failure

Under apoptotic stimuli, cells undergo programmed cell death generally through the extrinsic pathway, also called the death receptor pathway, and the intrinsic pathway, also named the mitochondrial pathway [1]. In general, extrinsic pathways are activated by the death receptors through the interaction between their natural ligands or by inducing death receptor clusterization. Death receptors belong to the tumor necrosis factor (TNF) superfamily and interact with their ligands to form death receptor complexes, including Fas (CD95/Apo1)/Fas Ligand (CD95 ligand) [15], TNF receptor 1 (p55)/TNF and lymphotoxin [16], TRAMP (WSL-1/Apo3/DR3/LARD)/TWEAK (Apo3 ligand) [17], TRAIL-R1 (DR4)/TRAIL (Apo2 ligand) [18], and TRAIL-R2 (DR5/Apo2/KILLER)/TRAIL [19]. Upon the activation of extrinsic pathway, the intracellular death domain (DD) of death receptors interacts with an adaptor protein Fas-associated death domain (FADD) directly or indirectly via the TNF receptor-associated death domain [19]. The FADD complex interacts with a typical initial procaspase-8 to form a death-inducing signaling complex required for the activation of caspase-8 [19]. Caspase-8 is able to cleave Bid to form a truncated form of Bid (tBid) and causes a reduction of mitochondrial transmembrane potential (MTP) followed by the release of cytochrome *c*, which binds to Apaf-1 and promotes caspase-9 and caspase-3 activation [20, 21].

In addition to the extrinsic pathway, the involvement of mitochondrial injury in apoptosis is proposed to be via an intrinsic pathway. Generally, activation of proapoptotic Bax and Bid, the members of the Bcl-2 family with proapoptotic roles, leads to its translocation to the mitochondria and disrupt the membrane integrity to induce MTP [22–24]. In contrast, Bcl-2 and Bcl-xL, the members of the Bcl-2 family with antiapoptotic roles, protect these effects by maintaining the MTP through the inhibition of Bax or other proapoptotic factors [22]. Dysregulation on the balance of Bcl-2/Bax contributes to the progression of apoptosis of intrinsic pathway.

Stresses on the endoplasmic reticulum (ER), which is the site of protein synthesis, modification, and folding, can trigger an unfolded protein response (UPR) following ER stress [25–27]. ER stress can be caused by the inhibition of glycosylation, the reduction of disulfide bonds, calcium depletion from the ER lumen, impairment of protein transport to the Golgi, and expression of mutated proteins in the ER. UPR of ER stress enhances protein folding and degradation within the ER and downregulates protein synthesis until cells have recovered from the ER stress. However, ER stress may also cause apoptotic cell death by the prolonged UPR and is demonstrated to be involved in several apoptotic signaling pathways [25]. The ER stress-induced transcription factor C/EBP homologous protein decreases the expression of antiapoptotic Bcl-2 and increases reactive oxygen species (ROS) production to trigger cell apoptosis through the mitochondrial pathway [26]. Apoptosis signal-regulating kinase (ASK) 1, an upstream kinase of c-Jun N-terminal kinase (JNK), is activated in cells through death receptors and oxidative stress [28]. ER stress can also activate ASK1. Deficiency on ASK1 reduces ER stress-induced JNK activation and cell death [29]. Cascade activation of caspases is generally involved in ER stress-induced cell apoptosis. Caspase-4, a specific ER stress-activated caspase with homology to murine caspase-12, triggers apoptotic pathways, dependent or independent of caspase-9 and caspase-3 activation [25]. The crosstalk between ER and mitochondria is therefore speculated. Meanwhile, the activation of caspase-2, -3, -7, -8, and -9 has also been reported in ER stress-induced apoptosis [30–32]. A feedback regulation of these caspases has been proposed to be mediated by ER stress-activated calcium-dependent protease calpain. Initially, activated calpain directly causes the activation of human caspase-4 [33, 34].

For cell death, acidic organelle lysosome plays a pivotal role in apoptosis and necrosis caused by oxidative stress, TNF-*α*, sphingosine, p53, and staurosporine [35, 36]. Mechanistic studies show that destabilization of lysosomal membrane and release of lysosomal content into the cytoplasm initiate the lysosomal apoptotic pathway. In general, mitochondrial/lysosomal crosstalk is regularly involved in cell death process. However, the precise lysosomal pathway in ER stress-induced apoptosis remains unclear. Signaling of apoptotic stimuli, such as calcium, ROS, ceramide, sphingosine, phospholipase, Bax, Bim, Bid, and caspase causes lysosomal membrane permeabilization (LMP) [36, 37]. After LMP, cathepsins, the lysosomal proteases, translocate to the cytosol and trigger apoptotic and necrotic pathways through Bid truncation, caspase activation, and mitochondrial damage [38]. Overall, it is speculated that both ER stress and lysosomal and mitochondrial destabilization may contribute to the initiation stage of apoptosis. Inducing mitochondrial pathway is the major proapoptotic actions of ceramide, meanwhile, ceramide also causes lysosomal and ER dysfunction to facilitate apoptotic process. The crosstalk among these organelles in ceramide-induced apoptosis varies in the context of cell types and stimulations. 

## 3. Sphingolipid Metabolism

Membrane sphingolipids, regulators for cell growth, death, senescence, adhesion, migration, inflammation, angiogenesis, and intracellular trafficking, are bioactive metabolites including sphingosine, ceramide, sphingosine-1-phosphate (S1P), and ceramide-1-phosphate (C1P) [2]. Ceramide, a sphingolipid with sphingosine backbone, is generated from diverse pathways, including newly *de novo* synthesis and hydrolysis of sphingomyelin or cerebrosides [8, 13, 39, 40]. For *de novo* synthesis, ceramide is produced by palmitoyltransferase-mediated interaction of serine and palmitoyl-CoA and then a series of metabolic reactions. Alternatively, extracellular stimulation usually induces hydrolysis of sphingolipids and sphingomyelin by sphingomyelinase (SMase) and cerebrosides—including galactosylceramide and glucosylceramide by cerebrosidase.

**Figure 1:**
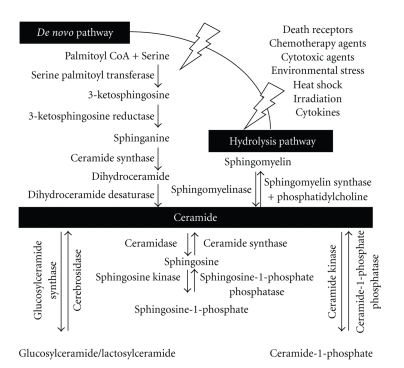
Metabolic pathways of ceramide. Under apoptotic stimuli, ceramide is generated primarily by *de novo *synthesis, through serine palmitoyltransferase- and ceramide synthase-mediated synthesis, and the hydrolysis of sphingomyelin through sphingomyelinase. Furthermore, metabolism of ceramide are regulated by ceramide kinase, sphingosine kinase, ceramide-1-phosphate phosphatase, sphingosine-1-phosphate phosphatase, cerebrosidase, and glucosylceramide synthase.

For the homeostasis of sphingolipid metabolism, ceramide is subsequently metabolized by ceramide kinase to generate C1P and by ceramidase to generate sphingosine, which is further phosphorylated to S1P by sphingosine kinase. Alternatively again, dephosphorylation of the metabolic derivates also occurs using specific phosphatases, such as C1P phosphatase and S1P phosphatase. Furthermore, ceramide can also be produced from sphingosine by ceramide synthase [8, 13]. As summarized in Figure 1, the dynamic regulation for ceramide generation and metabolism is critical for cellular responses to extracellular stimuli, such as death receptor-mediated (TNF-*α* and Fas), chemotherapeutic agent-mediated (etoposide, cisplatin, doxorubicin, paclitaxel, and inostamycin), and irradiation-mediated (UV and *γ*-irradiation) [12–14]. For tumorigenesis, ceramide acts as a tumor-suppressor lipid, whereas S1P acts as a tumor-promoting lipid [8].

A variety of sphingolipid metabolic disorders has been reported because of a deregulated balance on sphingolipid metabolism. Activation of S1P is essential for brain and cardiac development as well as for pathogenic in autoimmunity, cancer, and cardiovascular disease [4]. Ceramide, C1P, and S1P are able to facilitate activation of proinflammatory transcription factors in different cell types to induce overexpression of proinflammatory cyclooxygenase-2 (COX2) and prostaglandins [41]. Additionally, COX2 inhibitor celecoxib has been reported to induce apoptosis via activating ceramide *de novo* synthesis [42]. Deregulated ceramide facilitates the progressive neurodegenerative diseases such as Alzheimer's disease, Parkinson's disease, amyotrophic lateral sclerosis, and other neurological disorders that are characterized by the gradual loss of specific populations of neurons through the induction of neuronal cell apoptosis [7, 43].****

## 4. Proapoptotic Role of Ceramide

**Figure 2:**
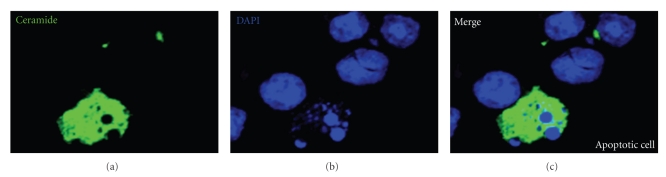
The generation of ceramide in apoptotic cells. Under high dose (25 mM) treatment of glucose (mimic hyperglycemia), mouse T hybridoma 10I cells underwent apoptosis were detected by 4′,6-diamidino-2-phenylindole (DAPI) nuclear staing. Fluorescein isothiocyanate-conjugated ceramide monoclonal IgM was used to detect ceramide generated in response to hyperglycemia.

Several lines of evidence have established the proapoptotic role of ceramide. Many apoptotic stimuli have been found to increase the levels of intracellular ceramide [12–14]. The role of ceramide had been speculated to be proapoptotic based on the observation that ceramide generation precedes the onset of apoptotic signaling [44], and exogenous treatment with ceramide induces cell apoptosis [45].  Figure 2 reveals the generation of ceramide in an apoptotic cell, typically with DNA fragmentation, under hyperglycemia treatment as detected by immunostaining using a monoclonal IgM against cemaride (clone MID 15B4). However, the causal relationships between ceramide generation and apoptosis had been in controversy for a while [46–48]. The advances in ceramide detecting methods and the discoveries of enzyme inhibitors to block ceramide synthesis or increase ceramide accumulation further identified that some ceramide-generating stimuli induce apoptosis in ceramide-dependent manner [12].

Ceramide is important mediator in both extrinsic and intrinsic pathways of apoptosis [6, 7, 12]. Endogenous ceramide generation and its roles in apoptotic signaling have been demonstrated in CD95- and TNF-*α*-treated conditions [49]. In these cells, acidic SMase mediates hydrolysis of sphingomyelin to generate ceramide and inhibition of acidic SMase effectively blocks cell death [50–52]. In addition to acidic SMase, TNF-*α* activates neutral SMase through FAN (factor associated with neutral SMase activation) to induced apoptosis [53]. Intrinsic apoptotic stimuli such as hypoxia, nutrient deprivation, radiation, heat, cellular stress, and cytotoxic drugs also increase endogenous ceramide levels through multiple mechanisms involving not only SMase but also ceramide synthase [2]. In general, increased ceramide causes activation of various protein kinases and phosphatases, cascade activation of caspases, dysfunction of multiple organelles, and leads to apoptosis [7].

Ceramide is structurally composed of a fatty acyl moiety bound to an amino alcoholic chain which varies in length from 2~28 carbons. Long-chain ceramide belongs to the natural form of ceramide found in cells. Short chain ceramides are usually synthesized for the purpose of research [54]. Obeid et al. [45] firstly identified the proapoptotic role of ceramide *in vitro* by using C2-ceramide (microM), a synthetic cell-permeable ceramide analog N-acetylsphingosine. Typically, C2-ceramide, but not dihydroceramide, induces internucleosomal DNA fragmentation, a characteristic for cell apoptosis [55]. In addition to C2-ceramide, C6-ceramide also triggers cell undergoing apoptosis [56]. After that, ceramide acts as a lipid second messenger as reported in a number of researches of apoptotic signaling pathways [57, 58]. In cancer cells, taking leukemia for instance, exogenous treatment of C2- and C6-ceramide induced apoptosis in chronic myeloid leukemia (CML) cell line K562 [59, 60]. C6-ceramide promotes apoptosis in CML-derived K562 cells by a mechanism involving caspase-8 and JNK [59]. Nanoliposomal delivery of exogenous ceramide (C6-ceramide) inhibit NK-LGL leukemia in a rat model and the antiproliferative effect of ceramide is through downregulation of antiapoptotic protein survivin [61].

Whether exogenous ceramide mimic intracellular physical actions of endogenous ceramide remains an open question. However, it is of note that treatment of cells with exogenous ceramide (at concentration below 20 microM to 2~10 × 10^6^ cells ml^−1^) resembles many cellular response induced by ceramide-generating stimuli [13]. Exogenous ceramide has been reported to induce endogenous ceramide through ceramide synthase [62]. Meanwhile, several interesting findings provide insight that the biologic functions of ceramide may vary by its length. Differential expression of C16- (high) and C18-ceramide (low) in patients with head and neck squamous cell carcinomas (HNSCCs) suggests the C18-ceramide play proapoptotic role but not C16-ceramide [63–65]. Knockdown of C16-ceramide synthase (ceramide synthase 6; CerS6) induces ER stress and apoptosis *in vitro* [66]. The growth of HNSCCs xenograft is promoted by overexpression of CerS6 but suppressed by overexpression of CerS1 (C18-ceramide synthase) [66]. The individual function of ceramide species needs more investigations.

Early evidence that Bcl-2 prevents ceramide-induced apoptosis [56, 67] demonstrates exogenous ceramide initiates mitochondrial apoptosis. In cell-free system, C2-ceramide induces ROS generation and inhibits mitochondrial electron transfer in isolated mitochondria [68–70]. C2- or C16-ceramide causes increase of mitochondrial outer membrane permeability which allows cytochrome *c* release [71, 72]. Ceramide can be generated in mitochondria through hydrolysis or *de novo* synthesis [73–75]. Both mitochondrial-overexpressed sphingomyelinase and TNF-*α* stimulation leads to mitochondrial ceramide accumulation, Bax translocation, cytochrome *c* release, and apoptosis [73, 76]. Exogenous C2- or C6-ceramide also triggers mitochondrial apoptosis in multiple cell lines [77–80]. In general, either exogenous ceramides or ceramide-generating death stimuli cause mitochondrial dysfunction, executor caspases activation, and apoptosis.

Compared with the field of mitochondrial apoptosis, much less studies address the role of ER in ceramide-induced apoptotic signaling. In addition to protein synthesis, ceramide is also *de novo* synthesized in the ER and transfer to Golgi apparatus by ceramide transport protein CERT [2, 3, 81]. The hypothesis that perturbation of ceramide level in ER might cause ER stress is deducible but needs more evidence. Knockdown of C16-ceramide synthase (CerS6) or C24-ceramide synthase (CerS2) induces ER stress [66, 82]. In glioma cells, tetrahydrocannabinol (THC) induces apoptosis through *de novo* synthesized ceramide-mediated p8 upregulation, which further trigger ER stress (induction of ATF4 and CHOP) [83]. Combination of histone deacetylase inhibitor vorinostat and multikinase inhibitor sorafenib induces *de novo* synthesized and acidic SMase hydrolyzed ceramide to upregulate CD95 to trigger ER stress (activation of PERK) [84]. CERT is identified to influence sensitivity of different cancer cell types to chemotherapeutic agents in a siRNA-based screening [85]. Downregulation of CERT sensitizes cancer cells to chemotherapeutic drugs and induces ER stress [85, 86]. Our previous report showed C2-ceramide and etoposide induce ER stress-related proteins Bip, CHOP, caspase-4, and PERK activation [87]. Requirement of ASK1 and JNK signaling is reported in both ceramide- and ER stress-induced apoptosis [28, 29, 87, 88]. Taken together, these findings suggest that ceramide induces ER stress; however, more investigations are needed to dissect the causal relationships and regulatory mechanisms between ER stress and ceramide-induced apoptosis. In addition, free fatty acid (FFA) has been reported to induce pathological ER stress and lead to metabolic disorders such as insulin resistance, obesity, steatosis, diabetes, and atherosclerosis [89–91]. The role of ceramide in insulin resistance has also been reported [92, 93]. Studies showed FFA activates ceramide *de novo* synthesis in islet *β* cells to induce apoptosis [94] and in astrocytes to increase A*β* protein expression and tau protein hyperphosphorylation [95]. However, another report demonstrated a ceramide-independent way of FFA to induce ER stress in liver cells [96]. More evidence is needed to better understand the regulation of ceramide in FFA-induced ER stress and downstream biological effects.

The mechanism by which ceramide triggers lysosomal apoptosis involves acid sphingomyelinase and cathepsin D [97–99]. Ceramide generated by acid SMase can directly interact with cathepsin D and mediate its activation [99–101]. In response to TNF-*α* stimulation, lysosomal acid SMase mediates ceramide generation to activate cathepsin D and downstream apoptotic signaling such Bid truncation and mitochondrial dysfunction [97]. Similar pathway has been recently reported in treatment of glioma cells with chemotherapeutic drug gemcitabine [99]. Exogenous treatment with C2-ceramide triggers this pathway in 10I hybridoma and A549 lung adenocarcinoma cells, however; different mechanism might exist because inhibiting acid SMase did not reduce LMP and apoptosis (Huang et al., unpublished data). Moreover, one study reported FFA-induced LMP and apoptosis in liver cells independently of ceramide *de novo* synthesis [102]. Concerning the findings of FFA-induced ER stress, the signaling pathways of FFA-induced apoptosis might have ceramide-dependent and -independent routes in triggering ER and lysosome dysfunction.

**Figure 3:**
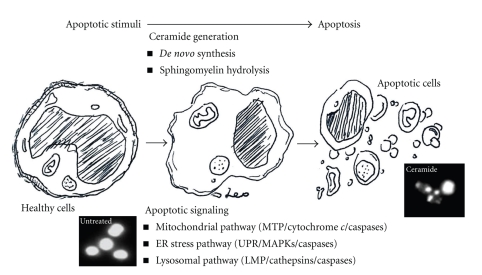
Proapoptotic ceramide. Apoptotic stimuli cause ceramide generation. Proapoptotic ceramide triggers apoptotic signaling through multiple mechanisms involving mitochondrial-, ER stress-, and lysosomal-regulated pathways.

In summary as shown in Figure 3, ceramide mediates dysfunction of multiple intracellular organelles followed by apoptosis. More investigations are needed to fully understand the cell type- and stimulus-dependent mechanisms of how ceramide causes organelles dysfunction and the crosstalk among these organelles. 

## 5. Regulation of Apoptotic Ceramide in Cancer Therapy Resistance

Cancer cells resist therapy in multiple mechanisms including escape from therapy-induced apoptosis [122–124]. Since the proapoptotic role of ceramide in cellular regulation is well established, either endogenous or exogenous ceramide contributes to the suppression of cancer progression [8, 14, 58]. On the other hand, cancer cells also gain survival advantages against therapy by impairing sphingolipids metabolism to reduce proapoptotic ceramide generation and accumulation [8, 9, 11, 125]. More than 30 enzymes are identified to regulate intracellular ceramide. Among them, glucosylceramide synthase, ceramidase, and sphingosine kinase are recently found to be targets for cancer cells to avoid killing of treatment. Here we summarized the findings about their roles in regulation of cancer therapy resistance (also listed in Table 1). 

### 5.1. Glucosylceramide Synthase (GCS)

Relationships between GCS and chemoresistance have been reported in various kinds of cancer cells and addressed in breast cancer most completely. In studies to compare the lipid compounds of drug-sensitive and -resistant cancer cell lines, glucosylceramides accumulation are found in drug-sensitive cells [126–129]. Ectopic expression of GCS increases chemoresistance of drug-sensitive cells. Reciprocally, genetic silencing and pharmacological inhibition of GCS sensitizes drug-resistant cells to multiple chemotherapeutic drugs, such as adriamycin, *Vinca* alkaloids, doxorubicin, etoposide, and paclitaxel [103, 104, 107, 130]. A recent *in vivo* study reported a mixed-backbone oligonucleotide against GCS sensitizes xenograft of multidrug-resistant breast cancer cell to doxorubicin [108]. GCS links multidrug resistance by multiple mechanisms, including reduced concentration of C18-ceramide, increased glycosphingolipids accumulation, and *MDR* gene upregulation through *cSrc *and *β*-catenin [104, 107–109, 130]. Upregulated *MDR* gene in turn encodes P-glycoprotein (P-gp), a drug efflux pump which induces multidrug resistance (MDR) in cancers [131–133]. One study reported that GCS-deficient and -ectopic expressed murine melanoma cells show no difference in the sensitivity to doxorubicin, vinblastine, paclitaxel, cytosine arabinoside, or short-chain ceramide analogs [134]. Another study showed that combining GCS inhibitor enhances doxorubicin-induced ceramide accumulation and apoptosis in hepatoma cells by a P-gp-independent manner [110]. These findings suggested that regulation of GCS in chemoresistance depends on cell type and might act through different mechanism.

Besides in breast cancer cells, overexpression of GCS is also found in multidrug-resistant leukemia, melanoma, colon cancer, and head and neck epidermoid carcinoma [104]. For example, in acute myeloid leukemia (AML), the ceramide levels are lower and the activities of GCS and sphingomyelin synthase are higher than in chemosensitive patients. The role of GCS is further confirmed in cell model when overexpression of GCS raised the resistance of HL-60 to doxorubicin-induced apoptosis [105]. In CML, drug-resistant K562 cells also express higher level of GCS. Pharmacological inhibition using PDMP or genetic silencing of GCS sensitizes drug-resistant K562 cells to adriamycin [111].

GCS and glycosphingolipids are also speculated to involve immune escape and metastasis in cancer [135, 136]. Overexpression of GCS and high secretion of glycosphingolipids might prevent cancer cell from immune attack by T cells and antibodies [135]. Preincubation with GCS inhibitor PDMP reduced the metastatic ability of Lewis lung carcinoma 3LL cells injected in mice [136]. However, more investigations are needed. 

### 5.2. Ceramidase

According to the maximal enzymatic activity in acidic, neutral, and alkaline environment, ceramidases are divided into acid, neutral, and alkaline ceramidases. Due to its ability to breakdown ceramide to regulate sphingosine and S1P levels, ceramidases become important regulators in cell survival [137]. Acid ceramidase is overexpressed in prostate cancer [138]. Ectopic expression of acid ceramidase in prostate cancer cell line DU145 shows elevated resistance to doxorubicin-, cisplatin-, etoposide-, gemcitabine- or C6-ceramide-induced apoptosis, while silence of acid ceramidase lowers the resistance to those drugs [112]. Acid ceramidase inhibitor B13 induces apoptosis in prostate cancer cell line and xenograft [139, 140]. In colon carcinomas, ceramide levels are lower than healthy tissue [141] and treatment with exogenous ceramide or ceramidase inhibitor B13 induces apoptosis [141, 142]. Acid ceramidase overexpression prevents fibrosarcoma cell line L929 from TNF-*α*-induced apoptosis and treatment of exogenous ceramide or acid ceramidase inhibitor *N*-oleoylethanolamine overcomes this TNF-*α* resistance [143]. Overexpression of neutral ceramidase prevents primary hepatocytes from TNF-*α*-induced apoptosis *in vitro* and inhibits D-galactosamine plus TNF-*α*-induced liver injury *in vivo* [144]. Another report showed high ectopic expression of alkaline ceramidase 2 in cervical cancer cell line HeLa leads to growth arrest due to sphingosine accumulation and low ectopic expression of alkaline ceramidase promote cell proliferation due to S1P production [145]. A recent study demonstrated silence of alkaline ceramidase 3 inhibits not only cell proliferation but also serum deprivation-induced apoptosis [146]. More evidence is needed to fully understand the regulation of neutral and alkaline ceramidases on the development of therapy resistance in cancer cells. 

### 5.3. Sphingosine Kinase (SphK)

Activation of SphK results in the generation of S1P to facilitate survival and therapy resistance in cancer cells. Ample evidence reveals the oncogenic role of SphK1; however, the isoform SphK2 seems to possess not only overlapping role with SphK1 in promoting tumor development but also opposite role in inducing apoptosis [147, 148]. Therefore, a recent study has developed a new SphK2 specific inhibitor [149] which might be used to further dissect the biological functions between the two isoforms. Overexpression of SphK1 promotes the development erythroleukemia [150]. In CML, which is caused by potent oncogenic protein Bcr-Abl, the activity of sphingosine kinase-1 (SphK1) is elevated by Bcr-Abl to increase expression of antiapoptotic protein Mcl-1 [151] and Bcr-Abl inhibitor imatinib-induced apoptosis through inhibiting SphK1 [152]. In myelodysplastic syndromes and acute leukemia, increased gene expression of SphK1 leads to doxorubicin resistance which in reverse can be abrogated by SphK1 siRNA [117, 153]. In solid tumors, SphK1 is required in the oncogenic signaling of vesicular endothelial growth factor (VEGF), epidermal growth factor (EGF), and Ras [154–156]. Overexpression of SphK1 has been identified in mRNA screening or immunohistochemistry staining in multiple cancer cells derived from breast, colon, lung, ovary, stomach, uterus, kidney, and rectum [157–159]. In human ovarian cancer cells, resistance to chemotherapeutic drug *N*-(4-hydroxyphenyl)retinamide (4-HPR) is mediated by SphK1 [114]. Prostate cancer cell line PC3 resistant to chemotherapeutic drug camptothecin is found to highly express SphK1 [118]. Higher activity of SphK1 and SphK2 in oxaliplatin-resistant colon cancer cell line RKO and knockdown of either SphK1 or SphK2 abrogates RKO cells oxaliplatin resistance [119]. One report showed hypoxia upregulates SphK2 protein expression as well as enzymatic activity to resist chemotherapeutic drugs-induced cell death in lung cancer cell line A549 [160]. 

### 5.4. Acid SMase, S1P Lyase, and CERT

Acid SMase-difficient MS1418 lymphoblasts from patients with Niemann-Pick disease show resistance to UVA and irradiation [161, 162]. An *in vivo* study also demonstrates mouse deficient in acid SMase showed resistance to irradiation-induced apoptosis [162]. Reciprocally, overexpression of acid SMase sensitizes glioma cells to gemcitabine and doxorubicin [120]. In cancer cells, many apoptotic stresses such as UV, irradiation, doxorubicin, cisplatin, TRAIL, and CD95 activates acid SMase to induce apoptosis [163, 164]. Activated acid SMase translocates to plasma membrane and increases ceramide to form ceramide-rich platforms (CRPs) which are required in signal transduction and apoptosis [165, 166]. One report suggested that the low fluidity of plasma membrane may be associated with cisplatin resistance [167]. However, whether cancer cells downregulate acid SMase to reduce CRPs formation and plasma membrane fluidity to achieve drug resistance remains unclear.

Opposite to the proapoptotic role of ceramide, S1P inhibits apoptosis by preventing release of mitochondrial cytotoxic effectors cytochrome *c* and Smac/DIABLO in several acute human leukemia cell lines [168]. Increasing S1P by downregulation of S1P lyase was found to alter S1P/ceramide rheostat to favor cell survival in colon cancer and ectopic expression of S1P lyase induces apoptosis via p53 and p38 MAPK [169]. Experimentally, using human embryonic kidney cells HEK293, ectopic expression of S1P lyase decreases cell viability and enhances ceramide generation and stress-induced apoptosis, meanwhile, addition of S1P reverses stress-induced apoptosis [170]. Overexpression of S1P lyase in HEK293 and A549 cells showed higher sensitivity to several chemotherapeutic drugs including cisplatin, carboplatin, and doxorubicin [121]. In addition, increased expression of ceramide transport protein CERT is found in residual tumor following paclitaxel treatment of ovarian cancer [85]. Loss of function of CERT leads to ceramide accumulation in ER and sensitizes cancer cells to chemotherapy and radiotherapy [85, 171]. 

## 6. Targeting Ceramide Metabolic Pathways to Overcome Cancer Therapy Resistance

Based on the knowledge that cancer cells develop resistance to therapy by arming themselves with the abilities to avoid generation/accumulation of intracellular proapoptotic ceramide, targeting ceramide metabolic pathways might be potential strategy to improve the response to cancer therapy. This strategy might itself be an alternative therapeutic option or in combination with present cancer therapies to enhance therapeutic efficacy and sensitivity.

Many chemotherapeutic drugs, such as daunorubicin, etoposide, camptothecin, fludarabine, and gemcitabine, have been known to induce ceramide *de novo* synthesis to mediate cytotoxic effects. Accordingly, it is conceivable that alteration of ceramide metabolism can significantly affect sensitivity of chemotherapy. GCS inhibition can restore sensitivity of drug-resistant cancer cells to multiple chemotherapeutic drugs [104, 106, 107]. The major cause of chemoresistance is therapeutic stress-induced P-gp overexpression which results in MDR. A set of MDR modulators, which bind and interfere with drug efflux, are used in combination of chemotherapeutic drugs to enhance efficacy [172]. MDR modulators sensitize drug-resistant cancer cells to chemotherapy through elevating ceramide levels by activating ceramide synthase or inhibiting GCS [173–176]. Combination of MDR modulators and GCS inhibitor synergistically induce cytotoxicity in various human solid tumor cell lines, including neuroblastoma and melanoma, prostate, lung, colon, breast, and pancreatic cancers [177]. SphK1 inhibitor by itself overcomes MDR-associated chemoresistance in AML and CML cell lines and patient samples and gemcitabine-resistant pancreatic cancer cells [115, 116, 178]. Downregulation of SphK1 or SphK2 enhances sensitivity to doxorubicin in breast cancer cell MCF-7 [179, 180]. One of the sphingolipid breakdown products, sphingosine, by itself induces apoptosis in adriamycin-resistant epidermoid carcinoma cells [181]. Inhibiting acid ceramidase with siRNA or *N*-oleoylethanolamine sensitizes hepatoma cells to daunorubicin [113]. Overexpression of alkaline ceramidase 2 but not alkaline ceramidase 1 or 3 enhances the cytotoxicity of 4-HPR in HeLa cells [182]. A recent report demonstrated combining exogenous C6-ceramide sensitizes multiple cancer cell lines to doxorubicin or etoposide [183].

For overcoming resistance to cancer radiotherapy, we summarize following examples. Defective ceramide metabolism causes resistance to radiation in AML and Burkitt's lymphoma cells [184, 185]. The defect-inducing proteins are needed to be identified for therapeutic target to improve radiosensitivity. Intracellular ceramide is also precursor of downstream prosurvival glycosphingolipids (precursor of gangliosides). Radioresistant sublines derived from human melanoma cell line M4Be are found rich in gangliosides. Combining fumonisin B1 (inhibitor of ceramide synthase) restore the sensitivity of radioresistant M4Be to radiation [186]. In glioma cells, combining either acid ceramidase inhibitor *N*-oleoylethanolamine or GCS inhibitor PDMP accelerates radiation-induced apoptosis [187, 188].

For overcoming resistance to cancer target therapy and gene therapy, we take CML and HNSCCs for examples. The Bcr-Abl tyrosine kinase inhibitor imatinib is the standard target therapy of CML [189]. Imatinib induces apoptosis in K562 cells via the generation of C18-ceramide, but this is not observed in imatinib-resistant cells [190]. Overexpression of CerS1 or silence of sphk1 enhances imatinib-induced apoptosis in imatinib-resistant cells [190]. Moreover, in our unpublished data, Bcr-Abl mutation-based imatinib resistance was abrogated by combining ceramide accumulating agents such as GCS inhibitor or ceramidase inhibitor, though in a not well-defined mechanism. Besides combinational treatment, C6-ceramide and SphK1 inhibitor independently induce apoptosis in imatinib-resistant CML cell lines KBM5 and LAMA84s, respectively [59, 152]. Sorafenib (BAY 43-9006) is a nonspecific inhibitor of RAF/MEK/ERK pathway and receptor tyrosine kinase [191]. Combining sorafenib with either SphK2 or SphK1/2 inhibitor provides enhanced growth inhibition of human pancreatic adenocarcinoma and kidney carcinoma cells *in vitro* and *in vivo* [192]. As prostate cancer, HNSCCs are also showed overexpression of acid ceramidase, therefore, acid ceramidase inhibitor is used to increase the cytotoxicity of adenovirus-delivered FasL in HNSCCs [193]. 

In addition to overcome cancer therapy resistance, targeting ceramide pathway might be a strategy to advance current conventional therapies into novel regimen on different cancer types. For example, sorafenib is approved for clinical use only in renal cell carcinoma [194]. Combining sorafenib with nanoliposomal ceramide enhances sensitivity to sorafenib in another cancer cell types including melanoma and breast cancer [195]. 

## 7. Conclusion

Cancer therapy resistance is a major problem leading to treatment failure. Mounting evidence indicated the apoptotic sphingolipid—ceramide—as important suppressor in cancer development. Alterations of ceramide metabolism become strategy for cancer cells to develop resistance against therapy. Conversely, manipulation of ceramide metabolism also provides potential alternative and combinational therapeutic options. Though, it is challenging to optimize solutions for overcoming resistance due to the complexity of sphingolipids metabolic network. By accumulating knowledge about how cancer cells escape from apoptotic stimuli and the discovery of potent and safe inhibitors, targeting ceramide metabolic pathways still provides opportunities for more feasible and more efficacious cancer therapy. 

## Figures and Tables

**Table 1:** Targeting ceramide metabolic enzymes alters drug resistance in cancers.

Enzymes	Cancer species (cell line)	Drug	Resistance	Reference
GCS ↑	Breast cancer (MCF-7)	Adriamycin	↑	[103, 104]
	Colon cancer (SW620)	Adriamycin	↑	[104]
	Epidermoid carcinoma (KB-3-1)	Adriamycin	↑	[104]
		Vinblastine		
	Leukemia (HL-60)	Vincristine	↑	[104]
	Melanoma (MeWo)	Etoposide	↑	[104]
	Leukemia (HL-60)	Doxorubicin	↑	[105]

GCS ↓	Adriamycin-resistant MCF-7	Adriamycin	↓	[106]
	Adriamycin-resistant MCF-7	Vinblastine	↓	[107]
		Paclitaxel		
	Adriamycin-resistant MCF-7 and murine breast cancer (EMT6)	Doxorubicin	↓	[108, 109]
	Adriamycin-resistant SW620	Doxorubicin	↓	[108]
	Doxorubicin-resistant ovarian carcinoma (A2780)	Doxorubicin	↓	[108]
	Doxorubicin-resistant cervical cancer (KB-A1)	Doxorubicin	↓	[108]
	Hepatoma (HepG2)	Doxorubicin	↓	[110]
	Multidrug-resistant leukemia (K562/A02)	Adriamycin	↓	[111]

Acid Ceramidase ↑	Prostate cancer (DU145)	Doxorubicin	↑	
		Cisplatin		[112]
		Etoposide		
		Gemcitabine		

Acid Ceramidase ↓	Hepatoma (HepG2, Hep-3B, SK-Hep and Hepa1c1c7)	Daunorubicin	↓	[113]

SphK1 ↑	Ovarian cancer (A2780)	4-HPR	↑	[114]
	Leukemia (HL-60)	Doxorubicin	↑	[115]
		Etoposide		
	Pancreatic cancer (Panc-1)	Gemcitabine	↑	[116]

SphK1 ↓	4-HPR-resistant A2780	4-HPR	↓	[114]
	Daunorubicin-resistant leukemia (K562)	Daunorubicin	↓	[117]
	Camptothecin-resistant prostate cancer (PC3)	Camptothecin	↓	[118]
	Oxaliplatin-resistant colon cancer (RKO)	Oxaliplatin	↓	[119]

Acid SMase ↑	Glioma	Gemcitabine	↓	[120]
		Doxorubicin		

S1P lyase ↑	Lung cancer (A549)	Cisplatin		
		Carboplatin	↓	[121]
		Doxorubicin		
